# Dasatinib and Trametinib Promote Anti-Tumor Metabolic Activity

**DOI:** 10.3390/cells12101374

**Published:** 2023-05-12

**Authors:** Eric L. Bolf, Thomas C. Beadnell, Madison M. Rose, Angelo D’Alessandro, Travis Nemkov, Kirk C. Hansen, Rebecca E. Schweppe

**Affiliations:** 1 Division of Endocrinology, Metabolism, and Diabetes, School of Medicine, University of Colorado Anschutz Medical Campus, Mail Stop 8106, Aurora, CO 80045, USAthomas.beadnell@gmail.com (T.C.B.); madison.m.rose@cuanschutz.edu (M.M.R.); 2 Department of Biochemistry and Molecular Genetics, School of Medicine, University of Colorado Anschutz Medical Campus, Aurora, CO 80045, USA; angelo.dalessandro@cuanschutz.edu (A.D.); travis.nemkov@cuanschutz.edu (T.N.); kirk.hansen@cuanschutz.edu (K.C.H.)

**Keywords:** Src, MAPK, combination, dasatinib, trametinib, thyroid cancer, metabolism, metabolomics

## Abstract

Thyroid cancer is the most common endocrine neoplasm, and despite its overall high survival rate, patients with metastatic disease or tumors that resist radioactive iodine experience a significantly worse prognosis. Helping these patients requires a better understanding of how therapeutics alter cellular function. Here, we describe the change in metabolite profiles after treating thyroid cancer cells with the kinase inhibitors dasatinib and trametinib. We reveal alterations to glycolysis, the TCA cycle, and amino acid levels. We also highlight how these drugs promote short-term accumulation of the tumor-suppressive metabolite 2-oxoglutarate, and demonstrate that it reduces the viability of thyroid cancer cells in vitro. These results show that kinase inhibition profoundly alters the metabolome of cancer cells and highlight the need to better understand how therapeutics reprogram metabolic processes, and ultimately, cancer cell behavior.

## 1. Introduction

Thyroid cancer is the most-diagnosed endocrine malignancy, with over 50,000 new cases per year in the United States [1,2]. Additionally, the incidence of this disease is growing, and thyroid cancer is estimated to be the fourth most commonly diagnosed cancer by 2030 [3]. The standard of care for differentiated thyroid tumors is primarily surgical resection of the tumor, but can also include total thyroidectomy and treatment with radioactive iodine [4]. Although the majority of patients respond well to therapy and overall survival rates for differentiated thyroid cancers are high, patients with metastatic disease or radioiodine refractory tumors exhibit a far worse prognosis [5,6]. In dedifferentiated thyroid tumors, surgical resection is often palliative, and radioactive iodide is ineffective [7]. For these tumors, generalized chemotherapy is used, as well as targeted therapies that inhibit driver pathways. The two major pathways that drive thyroid cancer are alterations to the mitogen-activated protein kinase (MAPK) pathway (specifically mutations in the *BRAF* gene (v-raf murine sarcoma viral oncogene homolog B1) or members of the *RAS* gene family) and to effectors within the phosphatidylinositol-3-kinase (PI3K) pathway [8]. Currently approved targeted therapies act upon the MAPK pathway, with particular emphasis on BRAF and mitogen-activated protein kinase kinaseMEK1/2. Thus, an increased understanding of how therapeutics repress tumor growth is vital to improving the current standard of care.

MAPK signaling is known to regulate metabolic function, one of the hallmarks of tumorigenesis [9]. Examples of MAPK signaling altering metabolic function include the association between BRAF mutations and increased ketogenesis in melanoma [10], as well as the association between RAS-mutations and central carbon metabolism in pancreatic cancer [11]. Our laboratory has shown that the combined inhibition of MEK1/2 and the non-receptor tyrosine kinase Src, a driver of thyroid tumor progression [12,13,14], is synergistic [15]. Src also has a role in the regulation of metabolic processes, having been demonstrated to promote the Warburg effect through the inhibition of pyruvate dehydrogenase in breast cancer, and to directly phosphorylate hexokinases I and II to promote their enzymatic activity [16,17]. Importantly, blocking the Src-mediated phosphorylation of hexokinase I and II blunted xenograft tumor growth [17].

As thyroid cancers tend to be more resistant to traditional therapies that target specific oncogenic drivers, such as the BRAF inhibitor vemurafenib [18], we hypothesized that we may be able to uncover additional metabolic therapeutic targets that, when inhibited, may act as circuit breakers to prevent the reprogramming associated with resistance to many tyrosine kinase inhibitors. In addition, defining metabolic regulation in response to targeted therapies can provide important information in regard to how Src and the MAPK pathway cooperate to drive the metabolic dependencies that promote oncogenesis. For these studies, we chose trametinib to inhibit the MAPK pathway, as it is approved by the Food and Drug Administration (FDA), in combination with the BRAF inhibitor dabrafenib in thyroid cancer and melanoma, and dasatinib to inhibit Src, which is FDA-approved for use in chronic myeloid leukemia (CML) [19,20].

## 2. Materials and Methods

### 2.1. Cell Culture

The human anaplastic thyroid cancer cell lines Cal62 (Research Resource Identifier (RRID): CVCL_1112) and CUTC60 (RRID: CVCL_VM61) and the papillary cell line MDAT32, as well as immortalized human thyroid follicular epithelial Nthy-ORI cells, were grown in RPMI (Invitrogen, Carlsbad, CA, USA) supplemented with 5% fetal bovine serum (Hyclone Laboratories) at 37 °C at 5% CO_2_ and 100% humidity [21]. MDAT32 cells were generated by MD Anderson [22]. Nthy-ORI cells were obtained from the University of Colorado Cancer Center Cell Technologies Shared Resource. All cell lines were validated via short tandem repeat profiling using the Applied Biosystems Identifier kit (#4322288) in the Barbara Davis Center BioResources Core Facility, Molecular Biology Unit, University of Colorado Anschutz Medical Campus, and routinely monitored for Mycoplasma contamination using the Lonza Mycoalert system (Lonza Walkersville, MD).

### 2.2. Metabolomics

Metabolite analysis was performed as previously reported [23,24]. Briefly, Cal62 cells were plated in duplicate at a density of 0.75 × 10^6^ in 10 cm plates and allowed to adhere for 24 h. Cells were then treated with either DMSO (dimethyl sulfoxide), 100 nM trametinib, 100 nM dasatinib, or their combination for 24, 8, or 2 h. For the cells collected for counting and Western blot analysis, cells were trypsinized (0.5 mL) and collected in 4.5 mL media, counted, and spun down at 1000 rpm for 5 min. The cell pellet was then lysed in 200 uL of NP40 lysis buffer + 1× protease and phosphatase inhibitor. The cells collected for metabolomics were harvested at 4 °C. Briefly, the medium was aspirated and cells were washed with cold phosphate-buffered saline (PBS). Lysis buffer was then added to normalize the lysates to 2 × 10^6^ cells/mL, and then, cells were scraped and collected. The lysates were then vortexed at 4 °C for 30 min. Lysates were then spun down at 10,000 rpm for 10 min, and then, the cell pellet and supernatant were separately frozen at −80 °C. For metabolite analysis, 2 × 10^6^ cells were extracted in a total volume of 1.0 mL lysis extraction buffer (methanol:acetonitrile:water; 5:3:2). After discarding the protein pellets, the water-soluble and methanol-soluble fractions underwent hydrophilic interaction liquid chromatography and were run through a C18 reversed-phase column using an ultra-high performance chromatographic system (Ultimate 3000, Thermo Fisher, Waltham, MA) at a 140,000 resolution (at 200 mass/charge). For metabolite assignment and peak integration quantitation, Maven software (Princeton) was used, and assignment was based on the Kyoto Encyclopedia of Genes and Genomes pathway database and an in-house-validated standard library (>650 compounds; SigmaIROA Tech, St. Louis, MO, USA). Principle component analysis (PLS-eDA) was performed using the GENE-E software (Broad), and heatmaps were generated using Morpheus (Broad). Box and whiskers plots were generated using GraphPad Prism V9 (Boston, MA, USA). Pathway analysis was conducted using the MetaboAnalyst web portal [25].

### 2.3. Cellular Assays

To measure cell growth, thyroid cells were treated with 0, 1, 5, and 10 mM dimethyl 2-oxoglutarate (Thermo Fisher, Waltham, MA, USA) for 72 h. Cells were then rinsed with PBS and trypsinized, and cell numbers were evaluated using a Vi-Cell XR Cell Viability Analyzer (Beckman Coulter, Indianapolis, IN, USA). Synergy was assessed after 24 h of treatment with 0.5, 1, and 2 mM dimethyl 2-oxoglutarate and 12.5, 25, and 50 nM each of dasatinib and trametinib. The viability of the cells was determined using a Cell-Titer Glo 2.0 assay (Promega) following the manufacturer’s directions. Excess over bliss was used to evaluate potential synergy. To assay apoptosis, cells were plated in white-walled 96-well plates and treated with 5 mM dimethyl 2-oxoglutarate for 24 h. Apoptosis was measured using a Caspase-Glo 3/7 Assay System (Promega) following the manufacturer’s directions.

### 2.4. Statistical Analysis

All data were tested for normal distribution using the D’Agostino–Pearson, Anderson–-Darling, Shapiro–Wilk, and Kolmogorov–Smirnov tests with alpha = 0.05 set as the cutoff for normality. For data that passed the normality test, a one-way ANOVA was performed, followed by Tukey’s multiple comparisons test for comparisons with multiple groups. For data that did not pass the normality test, a one-way ANOVA was performed, followed by a Kruskal–Wallis multiple comparisons test for comparisons of multiple groups. For comparisons between two conditions, a Student’s t-test was used to determine statistical significance. Significance was defined as *p* < 0.05, and variation is depicted as standard deviation. Synergy was calculated as excess over Bliss independence [26].

## 3. Results

### 3.1. Effects of Dasatinib and Trametinib on Metabolic Activity in ATC Cells

**Figure 1:**
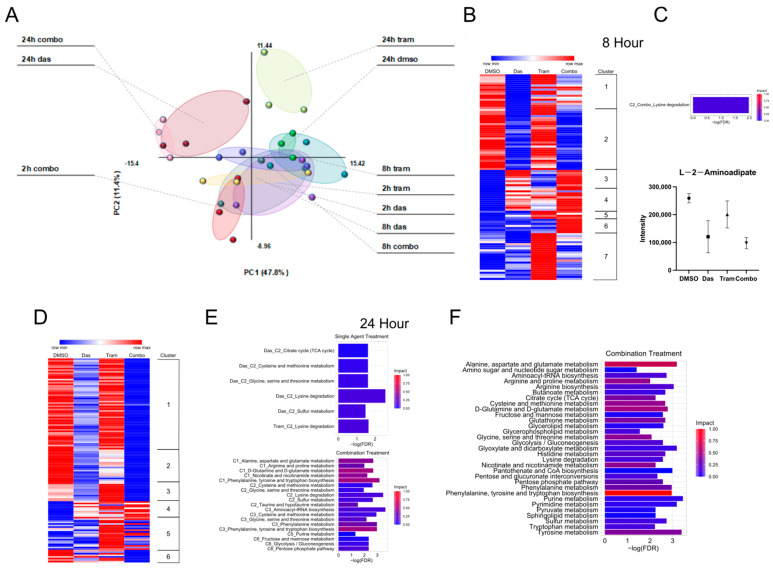
Dasatinib and trametinib treatments drive changes in metabolic activity. (**A**) Principal Component Analysis of metabolites in Cal62 cells after treatment with DMSO, 100 nM dasatinib (das), 100 nM trametinib (tram), or combination (combo) for 2, 8, and 24 h. (**B**) Changes in metabolite levels at 8 h were clustered and plotted on a heatmap. (**C**) Pathway analysis via MetaboAnalyst was performed on each cluster shown in B. (**D**) Metabolite levels at 24 h were clustered and plotted. (**E**) The clusters from D were analyzed using MetaboAnalyst. (**F**) Metabolites in the combo treatment when compared to DMSO were analyzed using MetaboAnalyst.

Cal62 thyroid cancer cells were treated for 2, 8, and 24 h with a vehicle (DMSO), 100 nM dasatinib, and 100 nM trametinib, or a combination of both drugs and metabolites were extracted and quantified via mass spectroscopy. We performed principal component analysis (PCA) on this dataset to broadly explore how these drug treatments altered global metabolite levels (Figure 1A). The greatest separation was observed between the DMSO group and the 24 h drug treatments. The 2 and 8 h treatment conditions largely clustered together. We next decided to focus on the 8 and 24 h time points for analysis. The metabolites for both of these time points were clustered by their patterns of regulation in order to make predictions about pathway regulation using the program MetaboAnalyst. As expected, the comparison between DMSO and 8 h drug treatments did not yield many pathways (Figure 1B). The sole pathway found to reach statistical significance, lysine degradation, was in the comparison between DMSO and the combination treatment, and its measured impact was minimal (Figure 1C). The analysis with the 24 h time point yielded more pathways (Figure 1D,E). The combination treatment resulted in even more statistically significant pathways, and the impact on those pathways was greater than that of the single-agent treatment. The pathways included multiple amino acid pathways as well as carbohydrate metabolism (i.e., the glycolysis/gluconeogenesis, fructose and mannose, and pentose phosphate pathways). When comparing the entirety of the metabolites between DMSO and the combination treatment, additional pathways emerged. These pathways also included multiple amino acid pathways and energy-yielding pathways (glycolysis and the tricarboxylic acid (TCA) cycle) (Figure 1F).

### 3.2. Combined Dasatinib and Trametinib Treatment Increases 2-oxoglutarate

**Figure 2:**
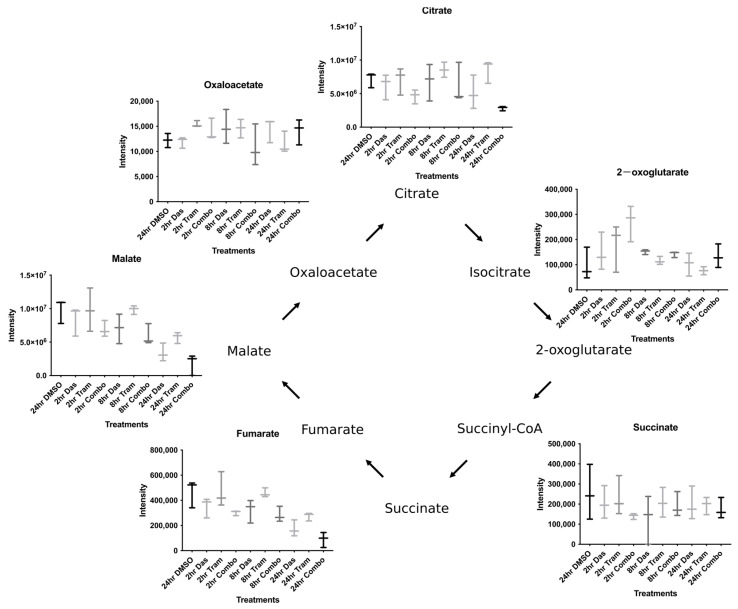
Levels of tricarboxylic acid pathway intermediates are altered by Src and MEK inhibition. Intermediates of the TCA pathway were pulled out from the metabolite dataset and quantified by intensity.

We first focused on the energy-yielding pathways of glycolysis and the TCA cycle. We observed a decrease in glycolytic metabolites in both the dasatinib- and combination-treated cells. Interestingly, we observed pronounced dasatinib regulation of the energy investment phase of glycolysis (Glucose 6-phosphateàFructose-1,6-Biphosphate), in which two ATP molecules are required for each molecule of glucose; this is consistent with the known regulation of hexokinase enzymes by Src [16,17] (Supplementary Figure S1). In contrast, we did not observe as much dasatinib regulation during the energy payoff phase of glycolysis (Bisphosphogluycerate à Pyruvate), in which two molecules of ATP are generated (Supplementary Figure S1). Instead, we only observed a substantial reduction in the energy payoff phase metabolites after 24 h of treatment with the combination therapy (Supplementary Figure S1). As the TCA cycle is central to oxidative metabolism and is directly connected to metabolism for many amino acids, we next decided to examine the metabolite levels of this pathway in greater detail (Figure 2). The metabolite 2-oxoglutarate, also referred to as α-ketoglutarate in the literature, piqued our interest as it was one of the few compounds that was increased after 2 h of drug treatment. Additionally, 2-oxoglutarate has been demonstrated to have anti-tumor properties [27,28]. Interestingly, the levels of neither glutamine nor glutamate increased following the rise in 2-oxoglutarate, and the levels of these amino acids did not decrease at the 2 h time point, suggesting that the increase in 2-oxoglutate was not a result of amino acid flux (Supplementary Figure S2).

Histone modification is an important process for the regulation of gene expression and is dysregulated in thyroid cancer cells [29,30]. Lysine demethylases require 2-oxoglutarate as a substrate to facilitate this process, and this may be a reason why we observed a loss of proliferation in the 2-oxoglutarate-treated cells. Methylation itself requires S-adenosyl-L-methionine (SAM), a component of one-carbon metabolism and, itself, a derivative of methionine. We observed that all drug treatments tested resulted in a marked reduction in SAM, which is strongly suggestive that epigenetic processes have been altered (Supplementary Figure S3). As 2-oxoglutarate has yet to be investigated in thyroid cancer, we decided to evaluate it in thyroid cancer cell lines.

### 3.3. 2-oxoglutarate Synergizes with Dasatinib and Trametinib to Inhibit Proliferation

**Figure 3:**
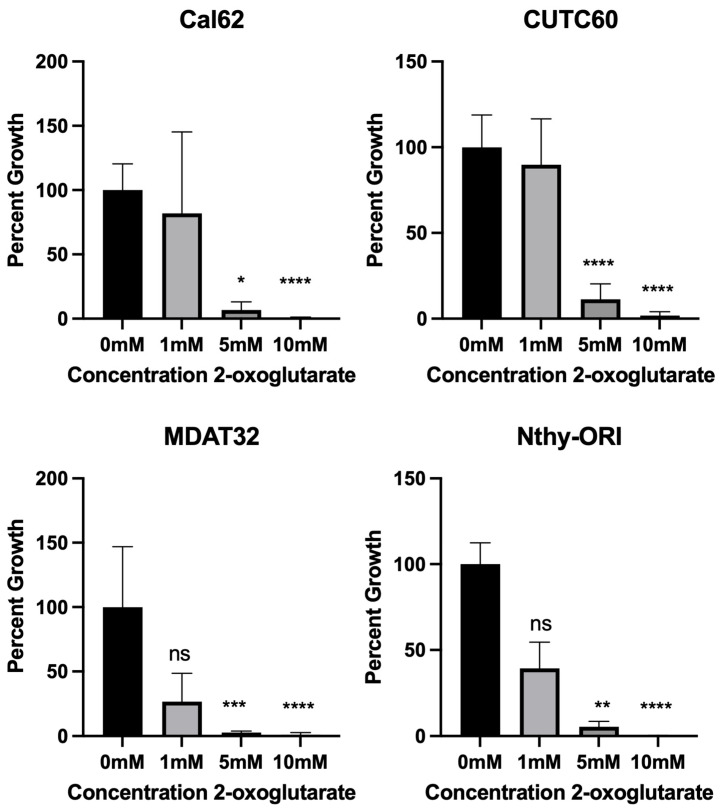
Increased levels of intracellular 2-oxoglutarate prevent cellular proliferation. Thyroid cells were treated with dimethyl 2-oxoglutarate, a membrane-soluble compound that is quickly metabolized to 2-oxoglutarate, for 72 h. Results shown are mean ± SD from three independent experiments performed in technical triplicate. Ordinary one-way ANOVA was performed in GraphPad Prism * *p* = 0.01, ** *p* = 0.0017, *** *p* = 0.0004, **** = *p* < 0.0001; ns = not significant.

**Figure 4:**
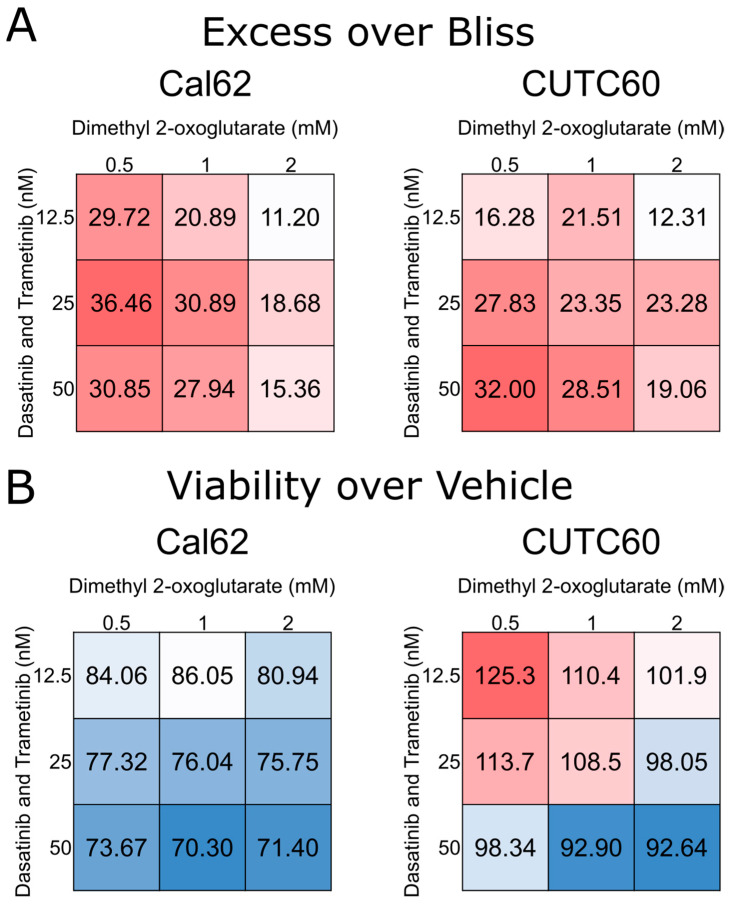
Low levels of dasatinib and trametinib exhibit synergistic activity with 2-oxoglutarate. Thyroid cancer cells were treated with a vehicle, dasatinib, and trametinib, and/or dimethyl 2-oxoglutarate, and viability was measured after 24 h. (**A**) Synergy was determined using excess over Bliss, where values > 1 indicate synergy and those <1 indicate antagonism. (**B**) Viability of the different treatment combinations indicated as percentages of the vehicle control. Results are compiled from 4 experiments with 3 technical replicates each.

In order to determine how 2-oxoglutarate affects thyroid cancer cells, we evaluated how it impacts cellular growth. Thyroid cancer cell lines were treated for 72 h with 0, 1, 5, and 10 mM dimethyl 2-oxoglutarate, a membrane-soluble compound that is quickly metabolized to 2-oxoglutarate, and cell growth was assayed (Figure 3). These doses were selected based upon in vitro studies in different cell types [31,32]. Unfortunately, the 10 mM dose yielded so few cells that cell counts were at or below the threshold of detection by the instrument. In all the thyroid cancer cell lines tested, we observed an anti-proliferative effect, although the Nthy-ORI cells, which were originally derived from healthy thyroid tissue, were also affected by the treatment. We also evaluated the cells for potential synergy between the combination of dasatinib and trametinib (considered a single agent) and 2-oxoglutarate (Figure 4). We observed synergistic activity between the two treatment modalities at the doses tested. These findings further support the notion that an intracellular 2-oxoglutarate from dasatinib and trametinib aid in the anti-proliferative action of these drugs.

## 4. Discussion

In this study we examined how cellular metabolites are changed following treatment with an Src inhibitor and a MEK1/2 inhibitor. We profiled changes to the cellular metabolome following 2, 8, and 24 h of pharmacological treatment, and performed pathway analysis. Interestingly, we did observe that dasatinib had a greater impact on metabolite levels than treating the cells with trametinib alone. This may be related to the known inhibitory effect Src inhibition has on hexokinases and the resulting perturbations to the glycolysis pathway. The additional potency may also be the result of Src regulating both the MAPK pathway and the PI3K pathway [15]. In particular, mammalian target of rapamycin (mTOR) is known to be an important metabolic regulator that is both regulated by and regulates amino acid levels [33]. Critically, multiple amino acid pathways emerged from the clusters of repressed metabolites. These are important raw materials for cellular growth and division, and blocking these pathways may be an important aspect of how dasatinib and trametinib repress tumor cell growth. The observed reduction in amino acids could have been the result of the dasatinib-mediated repression of mTOR action and resulting changes in the mTOR–amino acid feedback loop. Intriguingly, the suppression of mTOR activity is known to enhance the efficacy of dasatinib in other tumor types [34,35], and it may be worth investigating in the future whether this enhanced efficacy is the result of mTOR’s metabolic regulation or the metabolic changes are indicative of mTOR activity.

Other major changes noted included a significant reduction in amino acids and alterations to both glycolysis and the TCA cycle. These results suggest that the combination of inhibitors induces changes to the energetic balance of the cells. As cell proliferation is an energy-intensive process, this could reduce the ability of the cells to divide and progress through the cell cycle. Additionally, as the TCA cycle is a major source of raw materials for biosynthetic processes, alterations to TCA signaling may introduce new vulnerabilities to treated cells. We found that the TCA intermediate 2-oxoglutarate was transiently, but substantially, induced following treatment with these kinase inhibitors. This upregulation did not result in increased levels of either glutamate or glutamine, suggesting that it was not being shunted into amino acid pathways. Moreover, we did not find an increase in downstream TCA intermediates such as succinate or fumarate. We did find that treating thyroid cancer cell lines with membrane-soluble 2-oxoglutarate repressed cellular growth. 2-oxoglutarate has been found to phenocopy wild-type p53 transcriptional activity and is likely necessary for p53 signaling [31]. Interestingly, the phosphorylation status of p53 correlates with dasatinib resistance in melanoma cells [36]. Instead of supplying the amino acid or TCA pool, the heightened levels of 2-oxoglutarate may have instead been promoting growth suppression through p53.

In conclusion, this work details the metabolic alterations that occur in thyroid cancer following treatment with an MEK and an Src inhibitor. We have shown that dasatinib is more potent at reprogramming cancer cell metabolism and that energy-yielding pathways are altered, and have identified a metabolic intermediate, 2-oxoglutarate, that can reduce thyroid cell proliferation. This study sets the groundwork for determining how kinase inhibitors can alter the metabolic flux of thyroid cancer cells, and further research into the subject is necessary to develop novel treatment modalities that can exploit these changes.

## Data Availability

Data will be made available upon request.

## Supplementary Materials

The following supporting information can be downloaded at: https://www.mdpi.com/article/10.3390/cells12101374/s1, Figure S1: Glycolysis intermediates are repressed by dasatinib and trametinib; Figure S2: inhibitor-mediated increase in 2-oxoglutarate did not drive changes in glutamate or glutamine levels; Figure S3: Dasatinib and trametinib alter one-carbon metabolism.
