# Supplementary material for: Dasatinib and Trametinib Promote Anti-Tumor Metabolic Activity
Source: Cells. 2023 May 12;12(10):1374. doi: 10.3390/cells12101374 (PMC10216321; doi:10.3390/cells12101374)
Supplement: Supplementary file 1 [file cells-12-01374-s001.zip › cells-2320492-supplementary.pdf]

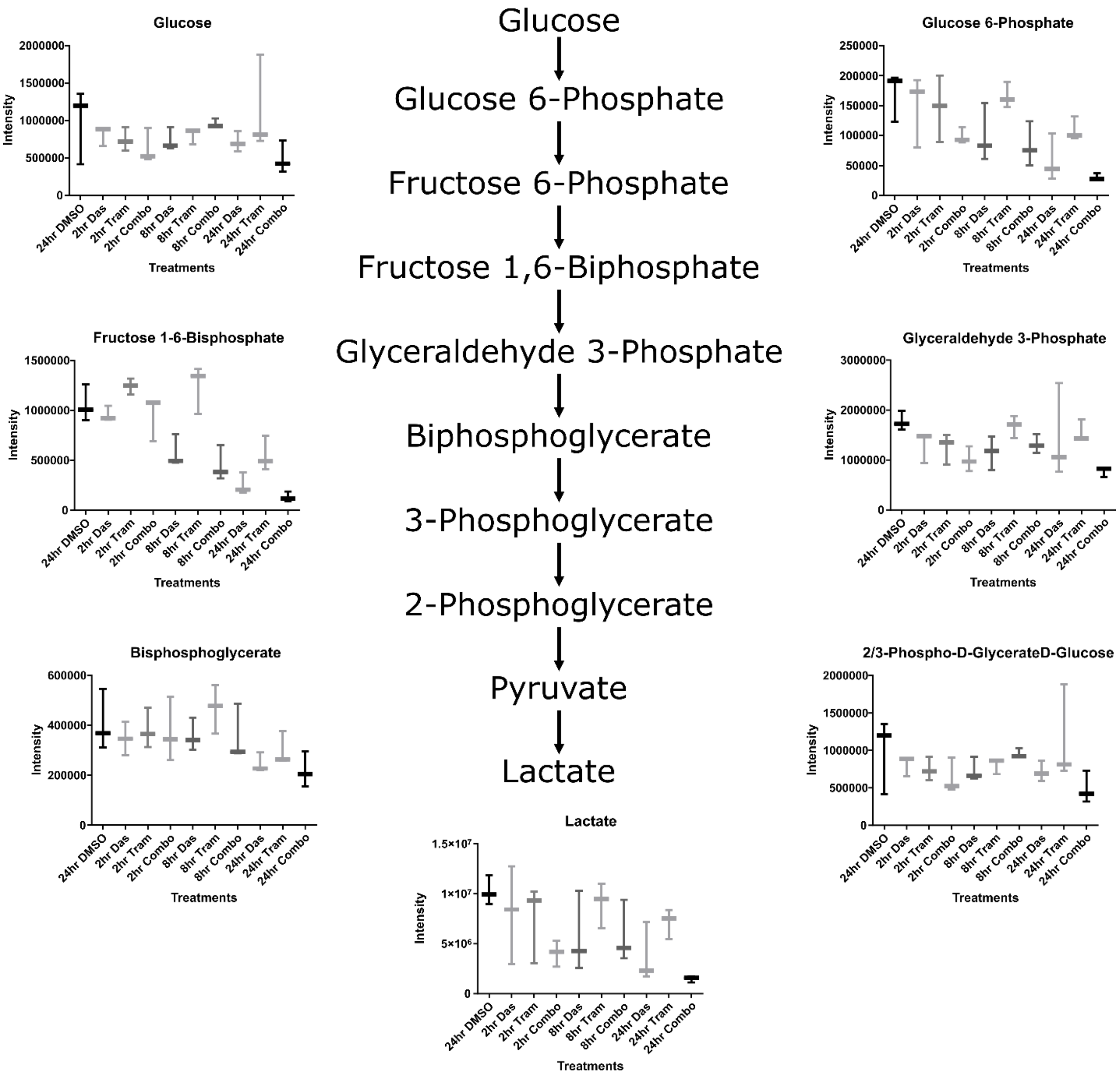

Supplementary Figure S1: Glycolysis Intermediates are Repressed by Dasatinib and Trametinib

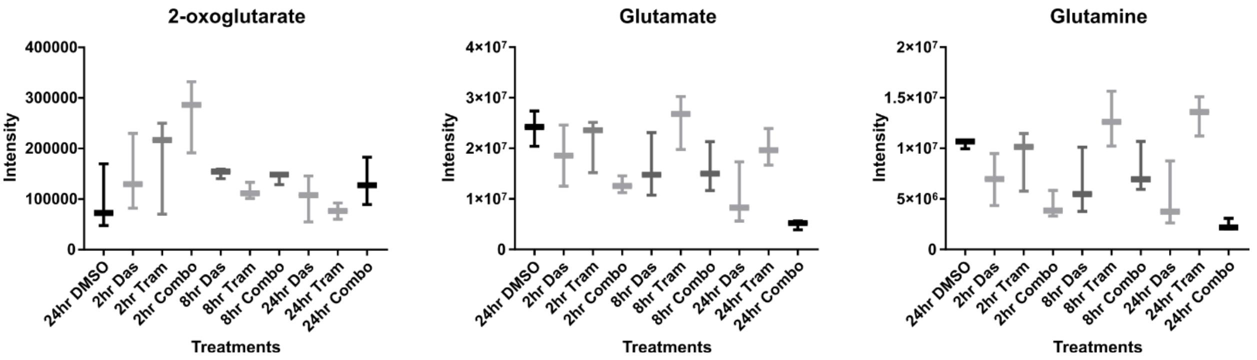

Supplementary Figure S2: Inhibitor Mediated Increase of 2-Oxoglutarate Did Not Drive Changes in Glutamate or Glutamine Levels

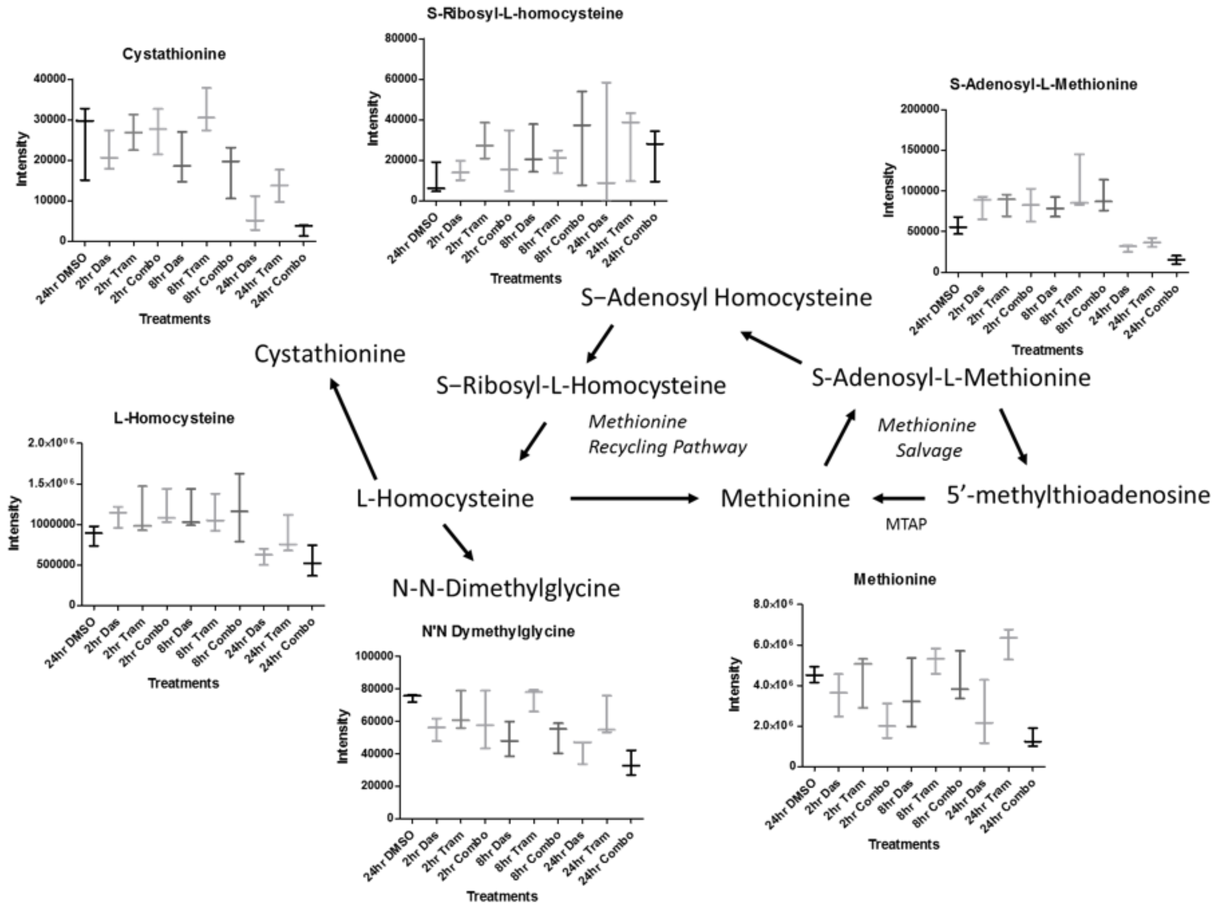

Supplementary Figure S3: Dasatinib and Trametinib Alter One-Carbon Metabolism
